# 16S rRNA Sequencing Reveals Alterations of Gut Bacteria in Hirschsprung-Associated Enterocolitis

**DOI:** 10.1055/s-0044-1789237

**Published:** 2024-08-22

**Authors:** Hao Shi, Yong She, Wu Mao, Yi Xiang, Lu Xu, Sanjun Yin, Qi Zhao

**Affiliations:** 1 Department of Surgery, Guangdong Women and Children Hospital, Guangzhou, China; 2 Department of Experimental Research, State Key Laboratory of Oncology in South China, Collaborative Innovation Center for Cancer Medicine, Sun Yat-sen University Cancer Center, Guangzhou, Guangdong, China; 3 Healthtimegene Institute, Shenzhen, China

**Keywords:** microbiota, diversity, metabolism, *Enterococcus*

## Abstract

Hirschsprung-associated enterocolitis (HAEC) stands as most common and serious complication of Hirschsprung's disease. Variations in the microbiota composition may account for the differences observed between HAEC and healthy individuals, offering crucial insights into the disease's pathogenesis. Here, we performed a study to changes in the gut microbiome using 16sRNA amplicon sequencing in a cohort of HAEC patients ( *n* = 16) and healthy controls ( *n* = 14). Our result revealed a significant disparity in beta diversity between the two groups. Following correction for false discovery rate, a rank–sum test at the genus level indicated a notable decrease in the relative abundance of *Bifidobacterium* , *Lactobacillus* , and *Veillonella* , whereas the *Enterococcus* genus exhibited a substantial increase in HAEC, a finding further supported by additional linear discriminant analysis effect size analysis. Functional analysis showed that putative transport and catabolism, digestive system, and metabolism of cofactors and vitamins were proved to be some abundant KOs (Kyoto Encyclopedia of Genes and Genomes [KEGG] orthologs) in healthy group, whereas infectious disease, membrane transport, and carbohydrate metabolism were the three KOs with the higher abundance in the HAEC group. Our data increased our insight into the HAEC, which may shed further light on HAEC pathogenesis. Our study firstly demonstrated the difference between fecal microbiota of HAEC patients and healthy individuals, which made a step forward in the understanding of the pathophysiology of HAEC.

## Introduction

Hirschsprung-associated enterocolitis (HAEC) is a potentially life-threatening complication that commonly arises in individuals with Hirschsprung's disease, spanning from the neonatal stage to adolescence. Its occurrence rates vary from 4 to 54%, with mortality rates ranging from 1 to 10%. 1 2 It is particularly concerning that although the frequency of HAEC tends to decline after the age of 2, a substantial number of children still remain susceptible to this life-threatening condition. 2 3

Bacteria widely exist on Earth as communities that consist of a massive number of bacterial species, and they often tend to coexist in symbiosis with other organisms. 4 The microbiome, which refers to the collective microbial population, plays a significant role in human health and disease, including the development of cancer. Researchers have proposed that individuals with Hirschsprung's disease possess a distinct fecal microbiota compared with healthy infants. 5 Previous studies conducted on mice with Hirschsprung's disease have shown that the alpha diversity of the intestinal microbiota increases over time and then decreases after surgery. 6 Altered mechanisms involved in maintaining gut homeostasis and immune system responses, attributed to genetic susceptibility or defects in epithelial cell barrier components, have been reported in Hirschsprung's patients who develop HAEC. 7 8 9 10 Several studies have observed differences in the composition of the microbiota between individuals with HAEC and those with non-HAEC. 6 11 12 13 However, there have no study in microbiota composition in HAEC compared with healthy individuals.

Conducting a thorough investigation comparing the gut microbiota composition in patients with HAEC and healthy individuals is of utmost importance for gaining deeper insights into the underlying pathophysiology of the disease. In this study, we employed 16S rRNA amplicon sequencing to analyze a cohort consisting of seven HAEC patients and eight healthy controls. Based on the dataset, a comprehensive bioinformatic analysis was performed.

## Materials and Methods

### Sample Collection

All studies were conducted with the approval of the Guangdong Women And Children's Hospital And Health Institute. Participants' intestinal contents, including 16 healthy controls and 14 HAEC patients, were sampled from the Guangdong Women and Children's Hospital and Health Institute. The study participants were minors, and all had parental consent. All participants' parents signed informed consent forms. The recruitment period started on September 28, 2021 and ended May 2024. The intestinal content specimens were placed on dry ice immediately after collection and transferred within 30 minutes to a −80°C freezer, where they were stored without any additive until analysis.

### 16S rRNA Gene Amplicon Sequencing

DNA from samples was extracted according to manufacturer's protocols based on cetyltrimethylammonium bromide/sodium dodecyl sulfate (CTAB/SDS) method (the E.Z.N.A. Soil DNA Kit; Omega Bio-tek, Norcross, Georgia, United States).

The V3–V4 region of the bacteria 16s rRNA genes was polymerase chain reaction (PCR)-amplified (98°C for 1 minute, followed by 30 cycles at 98°C for 10 seconds, at 50°C for 30 seconds, and at 72°C for 30 seconds, and a final elongation at 72°C for 5 minutes) from the extracted DNA using the primer pair 341F 5ʹ-CCTAYGGGRBGCASCAG-3ʹ and 806R 5ʹ-GGACTACHVGGGTWTCTAAT-3ʹ. 14 PCR reactions were performed in a 30 μL solution containing 15 μL of Phusion High-Fidelity PCR Master Mix, 2 μL of 2.5 mM dNTPs, 3 μL of each primer (5 μM), and 10 ng of template DNA. Samples with bright main strip between 400 and 450 bp were chosen for further experiments. PCR products were also quantitatively assessed using a Qubit 2.0 Fluorometer (Life Technologies, United States) and diluted according to Illumina's standard protocol for Sequencing on Illumina MiSeq (Illumina Inc.). The sequencing was performed by Health Time Gene Institute in Shenzhen, China.

### Bioinformatics Analysis of 16S rRNA Gene Amplicon Sequencing Data

#### Raw Data

There is a certain amount of low-quality data, which will interfere with the results of the analysis, so it is necessary to preprocess the offline data before further analysis, and the specific processing steps are as follows. (1) Data quality control: (a) set 30 bp as the window length, if the average quality value of the window is lower than 20, the read end sequence from the window is truncated, and the final read length is less than 75% of the original read length is removed; (b) remove linker contamination reads (default adapter sequence has a 15 bp overlap with read sequence, set to 15 bp, and allow three mismatches; (c) remove reads with N content greater than 10%; (d) remove low-complexity reads (the length of consecutive occurrence of ATGC in default reads ≥10, set to 10 bp). (2) Primer removal was performed on QC data using the software Cutadapt (v4.5). (3) Tags Connection and Tags Filter: (a) tags ligation: the reads after removing the primers are connected into a tag sequence according to the overlap relationship between the reads using software Flash, with a minimum overlap length of 10 bp and an allowable mismatch ratio of 0.25. (b) Tags quality filtering: the original tags of the spliced use the software fastp (v0.22.0) for tags low-quality filtering. (c) Tags de-chimera sequence: the tags obtained after the above processing are detected by the software VSEARCH and Silva database, and the chimera sequence is finally removed to obtain the final valid data.

Assembled tags, trimmed of primers, and barcodes were further checked on their rest lengths and average base quality. We clustered the quality-filtered sequences into operational taxonomic units (OTUs) at a sequence similarity threshold of 97% using VSEARCH. 15 OTUs were assigned and identified to taxa using the SLIVA (v132), GreenGene (v13.8), and Unite (v7) reference libraries. Analytical methods such as multiresponse permutation procedure (MRPP) and Anosim were employed to compare potential similarities and differences among community structures of different sample groups. Linear discriminant analysis effect size (LEfSe) 16 was conducted to identify genera differentially. The screening threshold for LEfSe analysis was *p* < 0.05 and linear discriminant analysis (LDA) score ≥ 2. PICRUSt 17 (Phylogenetic Investigation of Communities by Reconstruction of Unobserved states) v 1.1.0 was used to detect predicted functional differences in gut microbiota between two groups. The principle of PICRUSt is based on the full-length 16S rRNA gene sequences of bacteria with known genomes. It infers their common ancestral gene functional profiles and extrapolates these profiles to predict the gene functions of other unsequenced species in the Greengenes database. This process aims to construct predicted gene functional profiles for the entire phylogenetic spectrum of archaea and bacteria. Finally, the sequenced microbial community composition is “mapped” to the database, enabling the prediction of metabolic functions for the microbial community.

### Statistical Analysis

Statistical analyses used in this study included the Wilcoxon test as described in the Figure legends.

## Results

### Clinical Characteristics of the Participants

The samples in our study were categorized into two distinct groups: individuals with a documented history of HAEC (S) and a control group consisting of healthy individuals (H). HAEC was clinically defined as the presence of a distended abdomen accompanied by symptoms such as abdominal pain, diarrhea, or malodorous stools. We did not find any significant differences between the two groups in factors such as gender, age.

### Altered Microbial Composition and Diversity in Hirschsprung-Associated Enterocolitis Patients

**Fig. 1:**
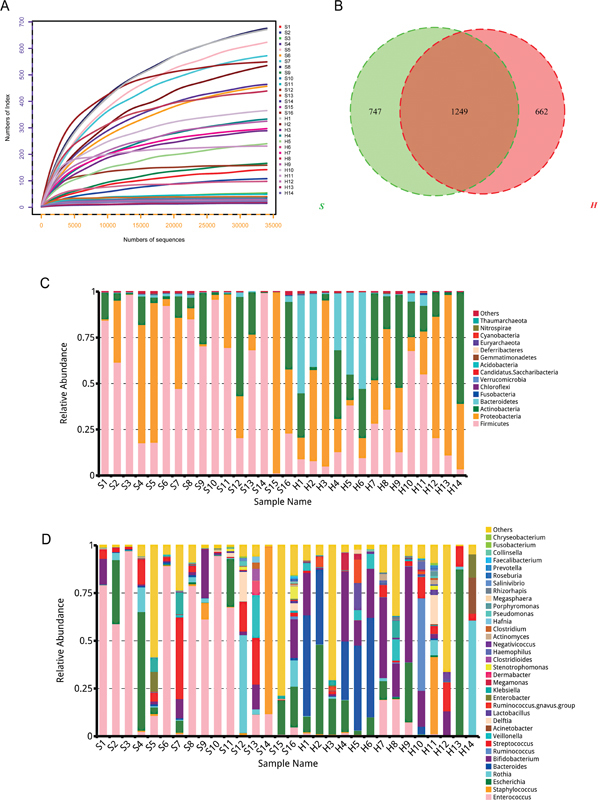
Microbial composition had a significant difference. ( **A** ) Rarefaction curves. ( **B** ) A Venn diagram displaying the overlaps between groups. A histogram displayed the relative abundance of the main phylum ( **C** ) and genera ( **D** ) in all samples.

The rarefaction curves indicate that the sequencing depth adequately captures the species information in the samples, meeting the analytical requirements ( Fig. 1A ). To explore the microbial diversity between patients with HAEC and healthy controls, we observed significant variation in the number of OTUs among samples, with the HAEC group showing slightly lower OTU counts compared with the healthy group ( Fig. 1B ). Subsequently, we conducted an analysis of the bacterial community composition between the two groups ( Fig. 1C, D ). In the S group, the dominant phylum was Firmicutes, whereas the H group exhibited higher relative abundances of the phyla Bacteroidetes and Actinobacteria compared with the S group. The top eight genera with higher abundances in the fecal samples from the different groups were *Enterococcus* , *Staphylococcus* , *Escherichia* , *Rothia* , *Bacteroides* , *Bifidobacterium* , *Streptococcus* , and *Ruminococcus* . Among these genera, *Bacteroides* and *Bifidobacterium* were the dominant genera in the H group, with considerably higher relative abundances compared with the S group. Conversely, the relative abundance of *Enterococcus* was significantly lower in the H group compared with the S group.

**Fig. 2:**
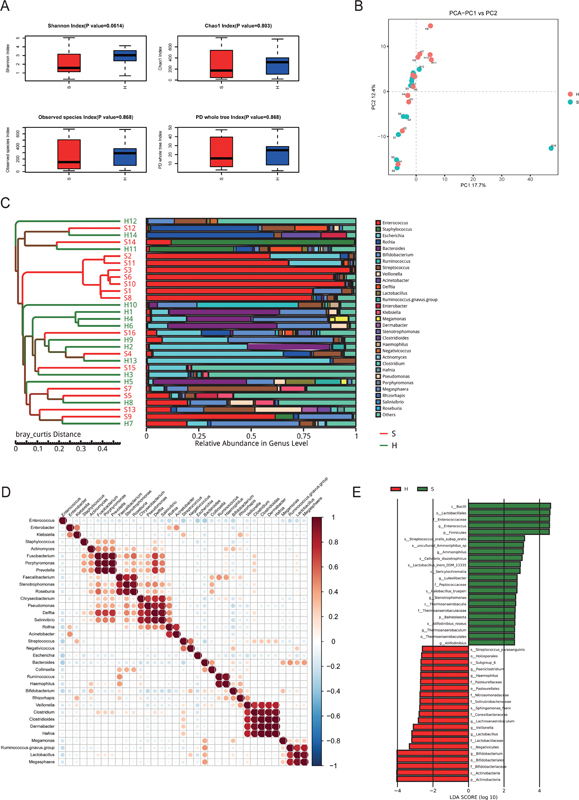
Taxonomic changes of gut microbiota at genus level between HAEC patients and healthy controls. ( **A** ) Compared with that in the controls, fecal microbial diversity, as estimated by Shannon index, Chao1 index, observed species index, and phylogenetic diversity whole tree Index. ( **B** ) Beta diversity was calculated using weighted UniFrac and were presented PCA. ( **C** ) A histogram displayed the relative abundance of the 30 main genera in all genera in all samples, simultaneously showing the clustering relationships among samples. ( **D** ) Genus level top 35 species correlation map display. ( **E** ) A histogram exhibits genera with significant effects on the division between groups, assessed by LEfSe analysis and corresponding influence represented as LDA score. HAEC, Hirschsprung-associated enterocolitis; LEfSe, linear discriminant analysis effect size; PCA, principal component analysis.

The fecal microbial diversity was assessed using the Shannon Index, revealing a notable decrease in the S group compared with the H group ( Fig. 2A , *p* = 0.0614). However, no significant differences were observed in other alpha diversity indices. To visualize the dissimilarity in the microbiome composition between the two groups, beta diversity was evaluated using unweighted UniFrac. The intergroup variance was further analyzed through principal component analysis (PCA), and statistical significance was determined using MRPP and Anosim analysis. The results demonstrated a marked difference between the two groups ( *p* = 0.01 for both MRPP and Anosim tests). The samples in the S group exhibited a significant aggregation, whereas those in the H group showed a scattered distribution ( Fig. 2B ). Additionally, a UPGMA cluster tree based on the weighted UniFrac distance also revealed distinct clustering patterns between the two groups ( Fig. 2C ).

### Taxonomic Changes of Intestinal Microbiota at Genus Level in Hirschsprung-Associated Enterocolitis Patients

To identify specific bacterial taxa associated with HAEC, we performed a comparison of fecal microbiota using the LEfSe. This analysis revealed several differentially abundant taxa at the genus level between the S group and H group. Notably, *Bifidobacterium* , *Lactobacillus* , and *Veillonella* were enriched in the H group, whereas *Enterococcus* showed a striking enrichment in the S group ( Fig. 2E ). *Bifidobacterium* is a commonly used probiotic, and recent studies have demonstrated its potential to attenuate major depressive disorder by regulating gut microbiome and tryptophan metabolism. Additionally, *Bifidobacterium* treatment has been associated with weight gain in Bangladeshi infants with severe acute malnutrition. *Lactobacillus* , another probiotic, has been found to enhance immune checkpoint blockade therapy, highlighting its potential benefits in respiratory diseases. 18

### Characteristics of Fecal Microorganism Interaction

It is intriguing to note that there may be correlations between different taxa based on their abundance changes. To assess the interaction relationships among the top 35 genera, we employed Spearman correlation analysis ( Fig. 2D ). Among these genera, *Faecalibacterium* , *Haemophilus* , *Ruminococcus* , *Akkermansia* , *Blautia* , and *Lachnoclostridium* exhibited a clear positive correlation, suggesting potential synergy among them. Conversely, *Bifidobacterium* , *Enterococcus* , *Pseudomonas* , and *Thiobacillus* displayed a negative correlation, indicating potential antagonistic interactions. These findings provide valuable insights into the possible interplay between bacteria, enhancing our understanding of the microbial ecology within the studied system.

### Function Characterization of Microbiome in Hirschsprung-Associated Enterocolitis Patients

Subsequently, our objective was to explore the functional profiles and differences in the metabolic potential of the gut microbiota in HAEC and healthy controls. A significant proportion of the overall genes in both groups were associated with metabolism, cellular processes, environmental information processing, and genetic information processing. This association was supported by depicting the relative abundance of comprehensive elements in each group at level 1 and level 2.

To assess the functional potential, we utilized Kyoto Encyclopedia of Genes and Genomes (KEGG) orthologs (KOs), categorizing proteins with highly similar sequences and similar functions along the same pathway. The abundance table of KOs was subjected to a rank–sum test to calculate the *p* -value for differences between two groups. KOs exhibiting significantly different expression between the HAEC group and the control group were identified based on a *p* -value threshold of <0.05. As a result, several KOs were found to be notably abundant in the healthy control group, including putative transport and catabolism, digestive system, and metabolism of cofactors and vitamins. Conversely, the HAEC group exhibited higher abundance of KOs associated with infectious disease, membrane transport, and carbohydrate metabolism ( Supplementary Fig. S1 , available in online version only).

## Discussion

Intestinal dysbiosis has been implicated in numerous diseases, 19 20 21 particularly inflammatory conditions. Patients with a Hirschsprung's disease exhibit a distinct intestinal microbiota composition compared with healthy individuals. However, the microbial composition differences between HAEC patients and healthy controls have remained unclear. In this study, our findings demonstrate a significantly altered microbial composition between the HAEC group and healthy controls.

Significant differences were observed in the composition of the microbiome, with the relative abundance of *Enterococcus* significantly lower in the H group compared with the S group. Conversely, the relative abundance of *Bacteroides* and *Bifidobacterium* was considerably higher in the H group compared with the S group. Fecal microbial alpha diversity, assessed by the Shannon Index, was notably decreased in the S group, aligning with findings in other disease reports. 22 Beta diversity, evaluated using unweighted UniFrac, revealed marked differences between the groups. PCA demonstrated a significant aggregation of samples in the S group and a scattered distribution in the H group. This observation was supported by statistical analysis, with significant results obtained from the MRPP and Anosim test ( *p* = 0.005 for both). Notably, *Bifidobacterium* , *Lactobacillus* , and *Veillonella* were notably enriched in the H group, whereas *Enterococcus* showed a striking enrichment in the S group. Importantly, *Enterococcus* are a part of human microbiota and a leading cause of multidrug-resistant infections. It can cause not only urinary tract infections, skin and soft tissue infections, but also life-threatening abdominal infections, sepsis, periostitis, and meningitis. 23 24 25 26 Studies have confirmed the pathogenicity of *Enterococcus* in recent years. Finally, we investigated the functional profiles and differences in the metabolic potential of gut microbiota in HAEC and healthy controls. The 16S sequencing data can only be accurate to the genus level, and the prediction of microbial function is not completely accurate, but it still has an important reference significance. Putative transport and catabolism, digestive system, and metabolism of cofactors and vitamins were proved to be some abundant KOs in healthy group, whereas infectious disease, membrane transport, and carbohydrate metabolism were the three KOs with the higher abundance in the HAEC group.

## Conclusions

It is interesting to note that the study has confirmed a significant change in beta diversity among HAEC patients. The findings of this study could potentially enhance our understanding of the pathogenesis of HAEC and help identify novel therapeutic targets based on manipulation of the microbiota. This highlights the importance of further research in this area to develop more effective treatments for HAEC.
