# Supplementary material for: 16S rRNA Sequencing Reveals Alterations of Gut Bacteria in Hirschsprung-Associated Enterocolitis
Source: Glob Med Genet. 2024 Aug 22;11(4):263–9. doi: 10.1055/s-0044-1789237 (PMC11341197; doi:10.1055/s-0044-1789237)
Supplement: Supplementary file 1 — Supplementary Material [file 10-1055-s-0044-1789237-s2400067.pdf]

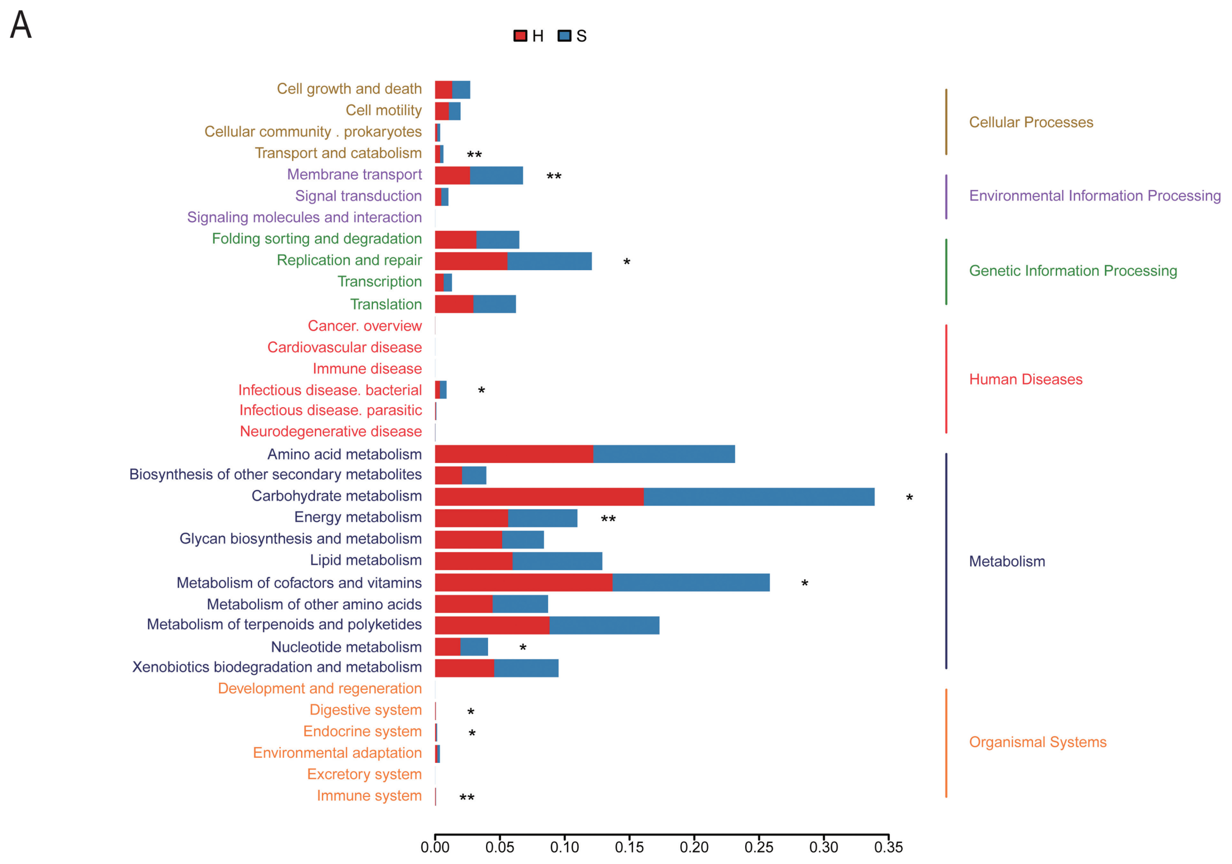

Supplementary Fig. S1 Metabolic pathway differences HAEC patients and healthy controls (A) Maps of species function and L1 and L2 levels of KEGG metabolic pathway in different groups of feces, the left of the figure represents the different secondary classifications of the KEGG metabolic pathway, the right represents the six classes of metabolic pathways that contain all metabolic pathways on the left.

 © 2024. The Author(s).
